# Development and validation of the functional assessment of cancer therapy–antiangiogenesis subscale

**DOI:** 10.1002/cam4.385

**Published:** 2015-01-26

**Authors:** Karen Kaiser, Jennifer L Beaumont, Kimberly Webster, Susan E Yount, Lynne I Wagner, Timothy M Kuzel, David Cella

**Affiliations:** 1 Department of Medical Social Sciences, Northwestern University Feinberg School of MedicineChicago, Illinois; 2 Department of Medicine, Northwestern University Feinberg School of MedicineChicago, Illinois

**Keywords:** antiangiogenesis, cancer, patient-reported outcomes, quality of life, side effects

## Abstract

The Functional Assessment of Cancer Therapy (FACT)–Antiangiogenesis (AntiA) Subscale was developed and validated to enhance treatment decision-making and side effect management for patients receiving anti-angiogenesis therapies. Side effects related to anti-angiogenesis therapies were identified from the literature, clinician input, and patient input. Fifty-nine possible patient expressions of side effects were generated. Patient and clinician ratings of the importance of these expressions led us to develop a 24-item questionnaire with clinical and research potential. To assess the scale's reliability and validity, 167 patients completed the AntiA Subscale, the Functional Assessment of Cancer Therapy-general (FACT-G), the FACT-Kidney Symptom Index (FKSI), the FACIT-Fatigue Subscale, the Global Rating of Change Scale (GRC), and the PROMIS Global Health Scale. Patient responses to the AntiA were analyzed for internal consistency, test–retest reliability, convergent and discriminant validity, and responsiveness to change in clinical status. All tested scales were found to have good internal consistency reliability (Cronbach's alpha 0.70–0.92). Test–retest reliability was also good (0.72–0.88) for total and subscale scores and lower for individual items. The total score, subscale scores, and all single items (except nosebleeds) significantly differentiated between groups defined by level of side effect bother. Evaluation of responsiveness to change in this study was not conclusive, suggesting an area for further research. The AntiA is a reliable and valid measure of side effects from anti-angiogenesis therapy.

## Introduction

Unlike chemotherapy, which treats cancer by killing cells, angiogenesis inhibitors block tumor blood vessel development. Side effects from angiogenesis inhibitors are generally milder than chemotherapy side effects, with 40–70% grade 1 or 2, based on National Cancer Institute Common Terminology Criteria grade 1. Nonetheless, these toxicities have the potential to impact health-related quality of life (HRQOL). Moreover, 10–20% of patients develop grade 3–4 toxicities that can result in dose reductions, delays, or discontinuation of therapy 1. Management of side effects is challenging for several reasons. First, side effect incidence and severity differ across drugs. Anti-angiogenesis drugs that directly target vascular endothelial growth factor (VEGF), a pro-angiogenic protein that increases tumor vasculature and metastatic growth 1,2, are associated with hypertension, proteinuria, and bleeding 2–6. Drugs which disrupt signaling through the multiple receptors for pro-angiogenic factors, the tyrosine kinase inhibitors (TKIs), produce fatigue, diarrhea, skin rash, stomatitis, reaction on the skin of hands and feet, hypothyroidism, and hematological abnormalities 2,7. Agents targeted to the mammalian target of rapamycin (mTOR), which is associated with increased angiogenesis by binding to intracellular protein (FKBP-12) 8, are associated with fatigue, rash, anemia, and metabolic abnormalities 9,10. Second, because these drugs are often lifelong therapies, both immediate and long-term toxicities are important concerns in patient management 1,2. Third, combining anti-angiogenic therapies or combing anti-angiogenic drugs with chemotherapy produces unique and sometimes unacceptable side effect profiles.

A standardized and validated measure to assess side effects of anti-angiogenesis therapies is needed to document profile differences and understand their relative importance. The development and validation of such a measure will allow for the collection of data to inform patient and provider treatment decision making. We aimed to develop and validate a brief, yet comprehensive, measure of side effects of anti-angiogenesis therapy, the FACT-AntiA.

## Methods

### Scale development: item generation and scale construction

#### Item generation

We conducted a comprehensive literature search to catalogue the side effects of anti-angiogenesis treatments. Semistructured interviews were conducted in-person or by telephone, in 2010–2011 with clinicians experienced in treating cancer patients with anti-angiogenesis therapy. Clinicians were asked to report the symptoms, side effects, and concerns of patients treated with anti-angiogenesis agents and rank these by importance to a patient's HRQOL. Semistructured interviews were also conducted with patients receiving anti-angiogenesis therapies who met these inclusion criteria: cancer diagnosis (any type, any stage); treatment with an anti-angiogenesis agent as monotherapy; age ≥18; and English-speaking. Patients were recruited from the Robert H. Lurie Comprehensive Cancer Center of Northwestern University. Written informed consent was obtained from interested study participants. Interviews were conducted by a trained interviewer in person or by telephone. Interviews identified the full range of HRQOL issues and concerns relevant to patients receiving anti-angiogenesis therapy. Patient participants were compensated $50 for their time and effort.

#### Scale construction

Data from the literature review and clinician and patient interviews were summarized to create an exhaustive and inclusive list of side effects of anti-angiogenesis therapy. Items representing these side effects were drawn from the Functional Assessment of Chronic Illness Therapy (FACIT) pool of more than 500 items 11. Additional items were drafted as needed and were written to be consistent with patients' descriptions of their side effects. A draft 22-item scale underwent cognitive debriefing with 10 patients currently on anti-angiogenic therapy. The cognitive debriefing protocol was based on the work of Willis et al. 12 and aimed to ensure that item content was understood as intended. Each item was coded 1 if the participant showed adequate comprehension of the item. Items were coded 0 if the participant misunderstood the item, misunderstood the response options, or expressed uncertainty regarding their response.

### Validation study: participants and procedures

The scale was validated in a sample of renal cell carcinoma (RCC) patients in 2011–2012. Eligibility criteria included: RCC diagnosis (any type, any stage), age ≥ 18; and English-speaking. The sample was stratified into three treatment categories: receiving an anti-angiogenesis agent, receiving other (non-anti-angiogenesis) therapy, and not currently receiving active therapy for their cancer. Those not currently receiving active therapy were further stratified according to whether they currently had no evidence of disease (NED). Participants for the validation sample were recruited by the Kidney Cancer Association (KCA; www.kidneycancer.org), a patient advocacy group for people with kidney cancer. Interested and eligible individuals were directed to Assessment Center^SM^ (AC), a secure, web-based survey administration and study management platform. Participants provided informed consent online and completed the study via self-report on AC. Participants received a $50.00 gift card for participating in the study. Participants completed assessments at baseline, 3–7 days later, and 8–12 weeks later.

### Validation study: measures

At time 1 (T1), participants provided sociodemographic information and self-reported clinical and treatment information, including the patient-reported Eastern Cooperative Oncology Group Performance Status Rating (ECOG-PSR). The ECOG-PSR includes the following response categories: normal activity without symptoms (0); some symptoms, but do not require bed rest during the day (1); bed rest for less than 50% of the day (2); bed rest for more than 50% of the day (3); unable to get out of bed (4). Participants also completed a battery of self-report HRQOL measures including the Functional Assessment of Cancer Therapy-General (FACT-G), which consists of 27 items in four domains (Physical Well-being [PWB], Social/Family Well-being [SWB], Emotional Well-being [EWB], and Functional Well-being [FWB]); the AntiA; the National Comprehensive Cancer Network (NCCN) FACT-Kidney Symptom Index-19 item (NFKSI-19); the FACIT-Fatigue Subscale; and the Patient-Reported Outcomes Measurement Information System (PROMIS) Global Health Short Form. At time 2 (T2), participants completed the FACT-AntiA (24 items). At time 3 (T3), participants completed the HRQOL measures administered at T1 plus the Global Rating of Change Scale (GRCS). The GRCS 13 is a 7-point scale (modified from the original 15-point scale) assessing change in well-being from “very much better” to “very much worse” in six domains: physical, social/family, emotional, functional, symptomatic, and overall quality of life. Patients provided updated clinical and treatment information at T3.

### Validation analyses

A total AntiA score was calculated by summing the responses on all 24 items. Prior to summing, all items were reverse scored such that a higher score corresponds to better HRQOL. Subscale scores for fatigue (three items), mouth sores (two items), hand/foot syndrome (four items), diarrhea (two items), and nausea (two items) were calculated by summing individual items scores. Internal consistency for the AntiA subscale scores and total score was evaluated using Cronbach's alpha. Test–retest reliability was evaluated for the AntiA subscales and total score between T1 and T2 using intraclass correlation coefficients (ICCs). Convergent validity analyses focused on differentiating definable (“known”) groups according to responses to the FACT-G item, “I am bothered by side effects of therapy.” Analysis of variance (ANOVA) was used to compare means between these defined groups. Effect sizes (mean difference/pooled standard deviation) were calculated to aid in interpretation of the group differences. To examine responsiveness to change, longitudinal data were used to calculate AntiA change scores (T1–T3) for each patient. Patients were categorized as improved, worsened, or unchanged according to their responses to the side effect bother item; mean AntiA total change was then calculated within each group. Spearman correlation coefficients were calculated to evaluate the association between change in the PRO scores and change in the anchor variables (side effect bother and ECOG-PSR). The standardized response mean was calculated as the mean change divided by the standard deviation of the change scores in that group. Paired t-tests were used to evaluate whether the change within a group was significantly different from zero.

## Results

### Generating important content from patients

We identified 527 articles related to anti-angiogenesis therapy. Seventy-six of these articles were deemed relevant to patient side effects, symptoms, and HRQOL; side effects mentioned in the articles were cataloged. Expert interviews were completed with 10 clinicians averaging 15 years of experience (range 3–28 years) treating cancer patients. Average age of the patient sample (*N* = 20) was 58 (range 34–75). Most (*N* = 13) of the sample was male. Sixteen (80%) had RCC; two (10%) had ovarian cancer, and two (10%) had brain tumors. The patients were receiving sunitinib (*N* = 13), bevacizumab (*N* = 6), or sorafenib (*N* = 1). The majority of patients (*N* = 18, 90%) were ECOG-PSR 0 or 1; two patients were ECOG-PS 2. Patient and clinician input led to the development of 59 preliminary scale items (items not shown here).

The study team reviewed the 59 items and removed redundant items and items inconsistent with patient descriptions of side effects, leaving 31 candidate items. The 31 candidate items were presented to the original panel of expert clinicians who rated each item according to relevancy to patients, prevalence of the symptom described, and ease of understanding. Review of clinician feedback led to the removal of nine items due to redundancy, poor wording, or irrelevance and to the rewording of two items. Clinician ratings of the resulting 22 items are shown in Table1; these items underwent cognitive debriefing to ensure they were clear to patients.

**Table 1:** Clinician (*N* = 10) ratings of the importance of symptoms to patient quality of life and patient cognitive interview results (*N* = 10)

Item	Clinician mean score^1^ 0 = not very important 1 = somewhat important 2 = quite important 3 = extremely important	Number of patients with good comprehension of the item
Hand pain or tenderness interferes with my daily activities	3.0	8
Pain on the bottom of my feet interferes with my walking	3.0	9
My fatigue keeps me from doing the things I want to do	2.8	10
I have to limit my activities because of diarrhea	2.6	10
I feel fatigued	2.4	9
I feel weak all over	2.4	9
I am bothered by hair loss	2.4	10
I have a loss of appetite	2.4	10
The skin on my feet hurts	2.2	10
Because of my mouth sores, eating is difficult	2.0	9
The skin on my hands hurts	2.0	9
I am bothered by a change in the way food tastes	2.0	10
I am bothered by a skin rash^2^	2.0	10
I have nausea	1.8	9
I am bothered by swelling in certain areas of my body	1.8	9
I have diarrhea	1.6	10
I am bothered by dry mouth	1.5	10
I am bothered by headaches	1.5	10
I am bothered by nosebleeds	1.4	10
I have pain in my joints	1.0	10
I am bothered by constipation	1.0	10
I am bothered by mouth sores or tenderness^3^	n/a	9
I have been short of breath^4^	n/a	n/a
I have been vomiting^4^	n/a	n/a

1 Clinicians were asked, “When present, how important is this symptom/issue to a patient's quality of life?”

2 Item was reworded from “I have a painful rash” following cognitive interviews to capture patient experiences with a wider range of rashes.

3 Physicians reviewed the item, “I have mouth sores,” which they ranked 1.25. This item was reworded to “I am bothered by mouth tenderness or soreness” for the cognitive interviews.

4 Item added following cognitive interviewing. Item drawn from the FACIT library and has previously undergone cognitive interviewing.

The cognitive interview sample (*N* = 10) were currently receiving bevacizumab (*N* = 5), sunitinib (*N* = 3), or temsirolimus (*N* = 2). Five participants were receiving anti-angiogenesis inhibitors as monotherapy; the remaining patients were receiving anti-angiogenic therapy in combination with chemotherapy. Mean age was 49 (range 27–77 years). The sample consisted of equal numbers of men and women. Cancers included RCC (*N* = 5), glioblastoma (*N* = 4), and non-small-cell lung cancer with brain metastases (*N* = 1). The majority of the patients were ECOG-PSR 2 (*N* = 6). Two patients reported no symptoms (ECOG-PSR 0); one reported some symptoms that did not require rest (ECOG-PSR 1) and one patient required rest more than 50% of the day (ECOG-PSR 3). Thirteen of the 22 items were comprehended by 100% of the participants (Table1). The remaining nine items were comprehended by 80–90% of patients. One patient, who disagreed with the word choice for most items, accounted for the majority of lack of understanding. Thus, the study team determined the overall comprehension of items was acceptable. Nine of 10 patients said the scale covered all side effects; one patient thought pain from medications should be included in the scale.

Following the cognitive interviews, the study team reviewed the scale. To ensure that symptoms of all anti-angiogenesis therapies were represented, two items from the preliminary set of 59 items—“I have been short of breath” and “I have been vomiting”—were added. These items were drawn from the FACIT library and have previously undergone cognitive debriefing with cancer patients. The final 24 items in the FACT-AntiA are highlighted in Table1. The AntiA was formatted according to a 5-point Likert scale (0, not at all; 1, a little bit; 2, somewhat; 3, quite a bit; and 4, very much).

#### Sample characteristics Validation Results

Of the 181 participants who enrolled in the validation study, 103 were not receiving treatment (57%), 64 were on anti-angiogenesis therapy (35%), and 14 were on other therapy (8%). Participants on other therapy were excluded from these analyses. Demographic, clinical, and key HRQOL characteristics for the remaining 167 participants are shown in Table2. Among participants on anti-angiogenesis therapy, the anti-angiogenesis drugs most often received were sunitinib (*n* = 27), everolimus (*n* = 12), and pazopanib (*n* = 9). NFKSI-19 scores for patients receiving anti-angiogenesis therapy were intermediate between the two groups of untreated patients (all *P* < 0.001). FACT-G scores, however, were similar to the NED group (*P* = 0.251) but better than the group with untreated disease (*P* < 0.001). All 167 participants completed the T2 assessment and 132 (79%) completed the T3 assessment.

**Table 2:** Descriptive statistics, validation study sample, Time 1 (*N* = 167)

	AntiA treatment (*n* = 64)	No treatment—disease present (*n* = 59)	No treatment—no disease (*n* = 44)	Combined sample (*n* = 167)
Demographics				
Age, mean (SD)	59.0 (7.3)	53.2 (10.8)	48.4 (8.0)	53.9 (9.8)
Female, *n* (%)	20 (31)	23 (39)	32 (73)	75 (45)
Hispanic, *n* (%)	2 (3)	2 (3)	0	3 (2)
White, *n* (%)	59 (92)	49 (83)	42 (95)	150 (90)
African American, *n* (%)	2 (3)	5 (8)	1 (2)	8 (5
Other race, *n* (%)	3 (5)	5 (8)	1 (2)	9 (5)
Patient-reported ECOG-PSR, *n* (%)				
Normal activity, without symptoms	12 (19)	21 (36)	31 (70)	64 (39)
Some symptoms, but do not require bed rest during waking day	39 (62)	29 (49)	13 (30)	81 (49)
Bed rest for less than 50% of waking day	11 (17)	9 (15)	0	20 (12)
Bed rest for more than 50% of waking day	1 (2)	0	0	1 (1)
Anti-angiogenesis therapy, n (%)				
Sunitinib	27 (42)	–	–	–
Everolimus^1^	12 (19)	–	–	–
Pazopanib	9 (14)	–	–	–
Sorafenib	6 (9)	–	–	–
Bevacizumab	5 (8)	–	–	–
Temsirolimus	2 (3)	–	–	–
Tivozanib (investigational compound)	2 (3)	–	–	–
Axitinib	0 (0)	–	–	–
Other	1 (2)	–	–	–
Current stage of kidney cancer, *n* (%)				
Stage 1	1 (2)	20 (34)	0	57 (34)
Stage 2	1 (2)	16 (27)	0	33 (20)
Stage 3	0	3 (5)	0	23 (14)
Stage 4	53 (84)	5 (8)	0	38 (23)
No disease	2 (3)	0	44 (100)	
don't know	6 (10)	15 (25)	0	13 (8)
Health-related quality of life, mean (SD)				
FACT-kidney symptom index	52.9 (9.8)	46.3 (11.7)	60.3 (9.6)	52.5 (11.8)
FACT-G	75.7 (15.9)	57.3 (17.9)	79.4 (17.0)	70.2 (19.4)

FACT, Functional Assessment of Cancer Therapy.

1 Everolimus, an mTOR inhibitor, has both immunosuppressant and anti-angiogenic properties, thus making its classification as an anti-angiogenic appropriate 14,15.

#### Reliability

For the AntiA total score and its subscale scores, the Cronbach's *α* exceeded 0.8 for all three subgroups and for the combined sample (range = 0.81–0.94). A coefficient above 0.7 is generally considered sufficient internal consistency reliability. Table3 shows mean AntiA scores and test–retest reliability of the AntiA total, subscales, and single items in the three subgroups and the sample overall. The test–retest reliability for the AntiA total was 0.88 in the combined sample. Subscale test–retest reliabilities exceeded 0.70 in the combined sample for all subscales. Reliabilities of single items ranged from 0.58 (shortness of breath) to 0.73 (several items).

**Table 3:** AntiA scores at baseline by treatment group and test–retest reliability (T1–T2) (*N* = 167)

	AntiA treatment (*n* = 64)	No treatment—disease present (*n* = 59)	No treatment—no disease (*n* = 44)	Combined sample (*n* = 167)	
	Mean (SD)	Mean (SD)	Mean (SD)	Mean (SD)	ICC
AntiA total (24-items)	75.2 (11.8)	65.7 (16.5)	86.6 (7.5)	74.8 (15.1)	0.88
Fatigue subscale (three items)	6.7 (3.4)	7.0 (3.2)	8.7 (3.0)	7.3 (3.4)	0.73
Mouth sore subscale (two items)	7.0 (1.7)	5.7 (1.9)	7.8 (1.0)	6.8 (1.8)	0.72
Hand/foot pain subscale (four items)	13.1 (3.2)	11.8 (3.4)	15.6 (1.1)	13.3 (3.2)	0.80
Diarrhea subscale (two items)	5.6 (2.5)	5.2 (2.2)	7.5 (1.3)	6.0 (2.3)	0.77
Nausea subscale (two items)	7.3 (1.3)	6.4 (1.5)	7.5 (1.0)	7.0 (1.4)	0.75
Single items					
Taste	2.9 (1.3)	2.9 (1.1)	3.7 (0.6)	3.1 (1.1)	0.72
Dry mouth	3.0 (1.2)	2.4 (1.2)	3.7 (0.5)	3.0 (1.2)	0.73
Headache	3.5 (0.8)	2.5 (1.2)	3.3 (0.9)	3.1 (1.1)	0.68
Joint pain	2.7 (1.1)	2.1 (1.1)	3.1 (1.2)	2.6 (1.2)	0.64
Constipation	3.6 (0.6)	2.9 (0.9)	3.5 (0.9)	3.3 (0.9)	0.69
Rash	3.4 (1.0)	2.7 (1.1)	3.8 (0.5)	3.3 (1.0)	0.73
Nosebleeds	3.7 (0.7)	3.2 (0.9)	4.0 (0.2)	3.6 (0.7)	0.64
Hair loss	3.5 (0.9)	2.5 (1.2)	3.8 (0.6)	3.2 (1.1)	0.73
Swelling	3.4 (1.0)	2.8 (1.1)	3.6 (0.8)	3.2 (1.1)	0.69
Appetite	3.2 (1.0)	2.6 (1.1)	3.6 (0.7)	3.1 (1.0)	0.69
Short of breath	2.7 (1.1)	2.7 (1.0)	3.5 (0.7)	2.9 (1.0)	0.58

ICC, intraclass correlation coefficient for test–retest reliability.

#### Discriminant (known-groups) validity

Table4 shows the differences in baseline AntiA scores by response to the item “I am bothered by side effects of therapy” in the total sample. All findings were generally in the expected direction—more side effect bother corresponded to lower AntiA scores (lower scores = poorer HRQOL). For example, participants who reported they were not at all bothered by side effects of therapy, on average, scored 84.8 on the FACT-AntiA. In contrast, participants who said they were very much bothered by side effects of therapy had a mean score of 58.2 on the AntiA (*P* < 0.001). Individual item comparisons indicated that these differences were statistically significant (*P* < 0.05) for all but the joint pain item.

**Table 4:** FACT-AntiA scores by side effect bother, Time 1 (*N* = 166)

		I am bothered by side effects of therapy					
		Not at all (*n* = 55)	A little bit (*n* = 51)	Somewhat (*n* = 37)	Quite a bit (*n* = 18)	Very much (*n* = 5)	*P* 1
AntiA	Mean (SD)	84.8 (13.5)	73.1 (12.7)	68.1 (12.5)	67.3 (14.3)	58.2 (16.6)	<0.001
	Effect size		0.88	0.40	0.06	0.62	
Fatigue	Mean (SD)	8.8 (3.1)	7.9 (2.2)	6.4 (3.0)	4.2 (3.8)	2.6 (3.4)	<0.001
	Effect size		0.35	0.56	0.66	0.44	
Mouth	Mean (SD)	7.7 (0.8)	6.4 (1.9)	6.1 (1.8)	6.9 (2.1)	4.8 (3.1)	<0.001
	Effect size		0.94	0.17	−0.44	0.89	
Hand/foot	Mean (SD)	14.9 (2.5)	12.8 (3.0)	11.8 (3.6)	12.8 (3.4)	12.2 (2.9)	<0.001
	Effect size		0.79	0.30	−0.28	0.17	
Diarrhea	Mean (SD)	7.2 (1.7)	5.3 (2.1)	5.3 (2.5)	5.4 (2.8)	5.6 (1.8)	<0.001
	Effect size		0.99	0.03	−0.05	−0.08	
Nausea	Mean (SD)	7.6 (0.8)	7.1 (1.3)	6.5 (1.7)	6.5 (1.7)	5.8 (1.5)	<0.001
	Effect size		0.51	0.38	0.01	0.42	
Taste	Mean (SD)	3.7 (0.7)	3.0 (1.0)	2.6 (1.2)	2.9 (1.4)	2.2 (1.3)	<0.001
	Effect size		0.85	0.35	−0.21	0.51	
Dry mouth	Mean (SD)	3.4 (1.0)	2.9 (1.0)	2.8 (1.1)	2.4 (1.5)	1.8 (1.3)	<0.001
	Effect size		0.48	0.17	0.29	0.39	
Headache	Mean (SD)	3.2 (1.1)	3.4 (0.7)	2.9 (0.9)	2.7 (1.5)	2.2 (1.5)	0.030
	Effect size		−0.18	0.56	0.15	0.35	
Joint pain	Mean (SD)	2.8 (1.3)	2.6 (1.1)	2.4 (1.0)	2.4 (1.3)	1.6 (1.7)	0.149
	Effect size		0.18	0.19	−0.04	0.61	
Constipation	Mean (SD)	3.5 (0.8)	3.3 (0.7)	3.4 (0.8)	2.7 (1.4)	3.6 (0.5)	0.020
	Effect size		0.26	−0.02	0.61	−0.67	
Rash	Mean (SD)	3. 7(0.7)	3.2 (1.0)	3.0 (1.2)	3.3 (1.1)	2.0 (1.0)	<0.001
	Effect size		0.63	0.14	−0.29	1.20	
Nosebleed	Mean (SD)	3.9 (0.4)	3.4 (0.9)	3.4 (0.8)	3.7 (0.8)	3.8 (0.4)	0.003
	Effect size		0.70	0.07	−0.39	−0.19	
Hair loss	Mean (SD)	3.7 (0.7)	3.0 (1.1)	3.0 (1.1)	3.2 (1.3)	2.8 (1.8)	0.007
	Effect size		0.74	−0.06	−0.17	0.30	
Swelling	Mean (SD)	3.5 (0.9)	3.2 (1.0)	3.1 (1.0)	3.1 (1.2)	1.8 (1.8)	0.004
	Effect size		0.37	0.09	0.05	0.92	
Appetite	Mean (SD)	3.6 (0.7)	2.9 (1.1)	2.9 (1.0)	2.4 (1.2)	3.0 (1.0)	<0.001
	Effect size		0.81	0.00	0.38	−0.46	
Short of breath	Mean (SD)	3.3 (0.9)	2.9 (0.9)	2.6 (0.9)	2.7 (1.1)	2.4 (1.5)	0.004
	Effect size		0.50	0.25	−0.02	0.22	

Effect size = (Difference in means)/(pooled standard deviation) for adjacent groups (i.e., Not at all vs. A little bit, A little bit vs. Somewhat, etc.). FACT, Functional Assessment of Cancer Therapy.

1 ANOVA *P*-value.

#### Responsiveness to clinical change

Table5 shows participant change over time (T1–T3) in side effect bother. The majority of participants (*N* = 75%, 57%) reported no change in their level of side effect bother. Similarly, their AntiA total score also did not change significantly from T1 to T3. Participants whose side effect bother score worsened by 1-point on the 5-point scale reported a significant decline in their AntiA score (*P* = 0.009). Participants in the other change categories (improvement or worsening) also had changes in AntiA scores in the expected direction, but these changes were not statistically significant. The correlation between FACT-AntiA change scores and change in side effect bother was 0.27. Correlations of individual item change scores with change in side effect bother (results not shown) ranged from −0.03 (mouth sores) to 0.31 (skin rash). Other items with correlations greater than 0.20 included dry mouth (0.21), swelling (0.25), and loss of appetite (0.23). Correlation with change in ECOG-PSR was −0.27 for PROMIS-Physical and −0.21 for PROMIS-Mental.

**Table 5:** FACT-AntiA by change in side effects bother and PROMIS Global change scores by change in ECOG-PS (*N* = 131)

“I am bothered by side effects of therapy”	*N*	Mean AntiA change (SD)	SRM	*P* 1
Improved by more than 1 point	10	4.6 (13.2)	0.35	0.30
Improved by 1 point	19	0.3 (10.7)	0.02	0.92
Unchanged	75	−0.5 (5.6)	−0.08	0.48
Worsened by 1 point	12	−6.2 (6.9)	−0.91	0.009
Worsened by more than 1 point	15	−3.7 (9.7)	−0.38	0.17
“I am bothered by side effects of therapy”	*N*	Mean AntiA change (SD)	SRM	*P*^1^
Improved by 1 or more points	29	1.8 (11.6)	0.15	0.42
Unchanged	75	−0.5 (5.6)	−0.08	0.48
Worsened by 1 or more points	27	−4.8 (8.5)	−0.57	0.007
ECOG-PSR	*N*	Mean PROMIS-Physical T score change (SD)	SRM	*P*^1^
Improved	13	4.2 (5.2)	0.80	0.01
Unchanged	86	0.1 (6.1)	0.02	0.87
Worsened	32	−3.3 (7.7)	−0.43	0.02
ECOG-PSR	*N*	Mean PROMIS-Mental T score change (SD)	SRM	*P*^1^
Improved	13	3.5 (6.1)	0.46	0.06
Unchanged	86	−0.4 (6.7)	0.06	0.57
Worsened	32	−2.7 (7.5)	−0.37	0.05

SRM, standardized response mean = (mean change)/(standard deviation of change scores); ECOG-PSR, Eastern Cooperative Oncology Group Performance Status Rating; FACT, Functional Assessment of Cancer Therapy.

1 *P*-value testing null hypothesis that mean change = 0 within group.

## Discussion

The FACT-AntiA, a novel, patient-centered assessment tool, was developed using patient and clinician input linked to relevant literature. The scale was developed to be “fair” across different anti-angiogenesis treatment options by adequately covering and balancing content to be included. After being developed, the questions underwent cognitive interviews with patients who overwhelmingly indicated that the scale items were comprehensible and captured their key concerns. The majority of participants in our scale development sample were receiving sunitinib or bevacizumab, thus current anti-angiogenesis therapies were not equally represented among our sample. However, we drew upon literature on all anti-angiogenesis side effects to guide scale development and obtained expert input on side effects from all therapies. The draft scale was reviewed to ensure representation of side effects from a range of therapies. The final scale includes items that represent the side effects from drugs not well represented in our sample. Our cognitive interview sample, which represented a more diverse range of therapies, overwhelmingly indicated that the scale contained the key side effects that were important to patients. Thus, the scale has good content validity. This property should make it more appealing to regulatory authorities and clinicians alike, who wish to be reassured that the questions are asking a representative set of concerns as experienced and expressed by patients themselves.

The AntiA subscale has good internal consistency reliability, with Cronbach's *α* exceeding 0.8 for the total scale and subscales. The scale and its subscales also exhibited good test–retest reliability. The AntiA scale, subscales, and individual items also demonstrated good discriminant validity–AntiA scores decreased (i.e., worsened) among participants who reported more bother with side effects. Our analyses of the scale's responsiveness to change indicate that the AntiA performed in the expected direction–AntiA scores improved as side effect bother decreased and deteriorated as side effect bother increased. However, there was little clinical change in our validation sample. Thus, our assessment of responsiveness to change should be interpreted with caution. Further study is needed to evaluate the instrument's responsiveness to meaningful change.

Scores on the FACT-AntiA for individuals with NED and individuals receiving therapy were in the expected direction: those receiving therapy reported more side effects. Thus, comparisons with the NED group support the validity of the FACT-AntiA. Participants with untreated disease scored lowest. We considered whether these participants had recently received therapy; only 10 of the participants with untreated disease reported receiving anti-angiogenesis therapies in the past. Three received anti-angiogenesis therapies within 90 days of study. Thus, recent therapy does not account for the group's low FACT-AntiA scores. Because this group also reported worse quality of life on the NFKSI-19 and the FACT-G, we hypothesize that their FACT-AntiA scores reflect overall poor quality of life and disease symptoms (e.g., swelling, shortness of breath, loss of appetite). Unfortunately, we are unable to confirm this hypothesis with our data. Comparisons with this group should be interpreted with caution.

All clinical data were self-reported, thus we were unable to verify treatments or disease status. Additionally, our data do not allow us to identify patients receiving sunitinib who were in the respite phase of dosing, which may impact symptoms. We expect that very few patients fell into this category. The geographic diversity of our validation sample—participants from around the United States (as well as a handful of international participants) completed the validation study assessments—is a strength of this study.

The FACT-AntiA contains subscales of key symptoms (fatigue, mouth sores, hand/foot, diarrhea, and nausea/vomiting). The number of items within each subscale was driven by empirical evidence for the relative importance of each of those areas. As a consequence, some multiitem subscales included two (mouth sores, nausea, diarrhea), three (fatigue), or four (hand/foot) items. Future initiatives should focus on the validation of the scale in other disease-types and should further evaluate the relative weighting of toxicities among patients receiving anti-angiogenesis therapies.

The FACT-AntiA may be useful in clinical practice and clinical research. In practice, one could explore the utility of this tool to identify specific anti-angiogenesis side effects and target them for treatment or symptom management. It can also be a trigger to identify when a treatment-induced symptom or symptom cluster is interfering with a patient's willingness or will to continue ongoing treatment. In clinical research, the subscale and total AntiA scores can be used to characterize anti-angiogenesis side effects and the extent to which they are changing over time, likely to be a more reliable estimate of change than one obtained, for example, by asking the patient informally or relying upon Common Terminology Criteria for Adverse Events (CTCAE). This would enable a fair patient-centered estimate of the extent to which one treatment is superior to another.
